# Psychosocial Predictors and Outcomes of Delayed Breast Reconstruction in Mastectomized Women in Mainland China: An Observational Study

**DOI:** 10.1371/journal.pone.0144410

**Published:** 2015-12-07

**Authors:** Yi Zhang, Hua Xu, Tao Wang, Jinguang He, Yufei Qiao, Jiao Wei, Jiasheng Dong

**Affiliations:** Department of Plastic and Reconstructive Surgery, Shanghai Ninth People's Hospital affiliated to Shanghai Jiao Tong University School of Medicine, Shanghai, China; Sudbury Regional Hospital, CANADA

## Abstract

**Background:**

The aim of the present study was to evaluate potential psychosocial factors that impact Chinese female breast cancer patients to select breast reconstruction (BR), and potential connection of psychosocial outcomes with their satisfaction with BR.

**Methods:**

A total of 264 female breast cancer patients with mastectomy were recruited from 2012 to 2014. All patients were informed with BR options at their first visit. Personal and medical profiles were collected. Body image, self-esteem, depression and anxiety were assessed using validated scales. Patients who were selected to undergo BR after the first visit were followed up for six months. The same assessment was performed at 6 months post BR, and their satisfaction with BR was evaluated using the Alderman scale. Multivariate linear and logistic regressions were performed.

**Results:**

Forty-seven percent of the patients (126/264) opted to undergo BR within six months after the initial visit. Multivariate logistic regression analysis revealed that self-esteem (P < 0.05), body image (P < 0.01), education level (P < 0.05), and their husband’s recommendation (P < 0.05) were highly related to the patients’ decision to undergo BR. In addition, multivariate linear regression analysis showed that patient satisfaction with BR was significantly associated with preoperative body image (P < 0.01), postoperative improvement in self-esteem (P < 0.01), improvement in body image (P < 0.01), reduction in depression (P < 0.05), pain (P < 0.05), and scarring (P < 0.01).

**Conclusions:**

The psychosocial factors including self-esteem and body image are highly related to selecting the BR option and post-BR satisfaction in Chinese female breast cancer patients.

## Introduction

Breast cancer (BC) has become the most common female cancer in Mainland China, with approximately 169000 new cases (17.8% of all cancer cases), accounting for around 10% of the global burden [1]. Mastectomy remains the first choice for more than 90% of Chinese BC patients [2]. For the majority of the survivors, significant contour deformities following breast amputation raise issues for psychosocial adaptation [3–5]. Breast reconstruction (BR) is an essential part of the treatment algorithm for BC, which offers effective long-term treatment to improve their psychosocial well-being [6,7]. In Western countries, the rates of all forms of BR among BC survivors range from 17 to 42 percent [8–11]. On the contrary, the BR frequency in Mainland China is estimated to be lower than 5%, although a rapid growth has been observed in recent years [12].

Patient decision-making for reconstruction is not entirely understood. Many previous studies have focused on socio-demographic predictors of BR including young age, high education, high income, being married, having insurance, and living in urban areas [13–18]. However, several psychological theories concluded that the decision-making process regarding female breast surgeries is more individualized and psychosocially influenced because of the symbolic meaning and psychological importance of this organ [19,20].

Keith and colleagues first hypothesized that mastectomized women who were more depressed because of breast loss had a higher tendency to undergo BR. [21] Similar findings were obtained by Duggal et al. in a sample of BC patients who were scheduled for mastectomy, depicting that fear of negative body image might serve as a motivation for BR [22]. These hypotheses have nonetheless only been partly confirmed. In these studies, only the intention of BR was documented from the participants but whether or not BR was conducted was unavailable. In addition, there are too few studies to allow multivariate analysis including other psychological variables involved in a possible relationship with BR. Considering that psychosocial functioning is inherently multifaceted, such studies are needed.

Patient satisfaction with BR is an issue of clinical interest. Objective assessment of aesthetic outcomes including breast size, shape, symmetry, skin color, and scarring, have been used in both Chinese and Western studies to determine the effectiveness of BR [23–25]. However, satisfaction from patient perspective is not on the basis of technical success of the surgery alone, but on a range of psychosocial factors and individual experience [26]. For example, there is already a consistent evidence that postoperative psychological functioning, such as body image, self-esteem, depression and anxiety were strong determinants of satisfaction with reconstructive surgeries in patient with facial deformity related to prior tumor resection [27,28]. However, the connection of these psychosocial functioning domains with the satisfaction experienced by BR patients remains less explored.

The aims of this study were: (1) to explore psychosocial predictors of delayed BR among BC survivors in Mainland China; (2) to examine postoperative psychological functioning including body image, self-esteem, depression, and anxiety in patients who underwent BR, and (3) to identify significant associations between these psychosocial functioning domains with patient satisfaction with BR.

To our knowledge, a prospective exploration of the psychosocial profile of Chinese female BC patients who select delayed BR after mastectomy, and the association between post-BR psychosocial changes with patient satisfaction has not been performed previously. We hypothesized that: (1) the presence of a negative psychological impact of breast loss was associated with the motivation for BR; and, (2) those who exhibited positive improved psychological functioning after BR would experience a higher level of satisfaction.

## Materials and Methods

### Ethic

The experimental protocol was approved by the Ethics Committee of Shanghai Ninth People's Hospital Affiliated to Shanghai Jiao Tong University, School of Medicine. The study protocol conformed to the Declaration of Helsinki. Participants gave written consent to participate after receiving an explanation of the procedures involved.

### Participants

A sample of 264 Chinese mastectomized women presenting to our outpatient clinics for a reconstructive consultation were consecutively enrolled from June 2012 to January 2014. Women were eligible if they were 18 years of age or older, had been treated with either a unilateral or bilateral mastectomy after diagnosis of BC, were not under adjuvant therapy or experiencing a recurrence, and had no other severe basic diseases. Exclusion criteria comprised development of malignancy in the contra-lateral breast, or the presence of distant metastases, or the occurrence of other major life changes at the time of survey which might affect psychosocial well-being.

### Procedure

Following the written patient informed consent, a 30-min self-report survey consisting of demographic, medical and psychological questionnaires was conducted. Indications, advantages, and complications of three types of BR (implant-based BR, implant plus autologous flap BR and autologous flap BR) were introduced by the same surgeon, and recommendations according to patients’ individual conditions were made. Women were categorized in the BR group if they opted to enter waiting list for the surgery, or categorized as no-BR group if they did not and had no plan for BR in the near future. Women receiving BR were followed at 6-months postoperatively, reassessed with the same questionnaires and a questionnaire that assessed satisfaction with BR. An e-mail interview was conducted when a face-to-face interview was not possible. All the BR were performed under general anesthesia by the same team as previously reported [24,29], at the Shanghai 9th People's Hospital.

### Measurements

#### Participant characteristics

Demographic and medical statistics including age, education, employment status, marital status, having children, household income, health insurance, affected side, time since mastectomy, and treatment of BC were obtained at the baseline visit. The husband’s attitude was collected if a woman was married or living in a marriage-like situation. Type of BR and the occurrence of major postoperative complications were gathered by direct medical record review. The maximum pain intensity experienced during the hospital period and the severity of the surgical scar at 6 months postoperatively were self-rated by patients, using a scale from 0 to 10 with 0 being the mildest and 10 being the most severe.

#### Self-esteem

The patients’ self-esteem was assessed using the Rosenberg Self-Esteem Scale (RSES), which is the most widely used and validated self-rated measure of self-esteem for the general population. The scale consists of 10 items, half positively stated and the other half negatively stated and reverse scored. Participants were asked to respond to each item using a 4-point Likert scale ranging from ‘strong agreement’ to ‘strong disagreement’. The scale produces a total score ranging from 10 to 40 points, in which a higher score indicates a higher level of self-esteem. Scores between 25 and 35 are within the normal range, and scores below 25 indicate low self-esteem [30].

#### Negative body image

The patients’ negative body image (NBI) was assessed using a 3-item subset of the Hopwood Body Image Scale. The items read: ‘feeling less feminine’, ‘feeling self-conscious (embarrassed) about your body ‘, and ‘worrying about your sexual attractiveness’. Participants were asked to respond to each item using a 3-point Likert scale ranging from ‘hardly ever or never’ to ‘much or most of the time’. The scale produces a total score ranging from 3 to 9 points, in which a higher score indicates a higher degree of body image dissatisfaction. Scores below 6 are within the normal range, and scores from 6 to 9 indicate a mild to severe body image disturbance. The reliability and validity of the scale for a BC population have been confirmed in previous research [31].

#### Depression and anxiety

The Patient Health Questionnaire nine-item (PHQ-9) and the Generalized Anxiety Disorder seven-item (GAD-7) tools were used to evaluate depression and anxiety level, respectively. The PHQ-9 assesses the DSM-IV criteria of depression. Participants were asked to respond to each of the 9 items using a 4-point Likert scale ranging from ‘not at all’ to ‘almost every day’. The attributed points for the PHQ-9 were added up to a total score of 0 to 27 points, scores of 5, 10, and 15 indicated mild, moderate, and severe depression symptoms, respectively. The answers and points of the GAD-7 questionnaire are analogous to the PHQ-9. The total score for the GAD-7 ranges from 0 to 21 points and scores of 5, 10, and 15 indicated mild, moderate, and severe anxiety symptoms, respectively. Both scales have shown strong reliability for the general population [32,33].

#### Satisfaction

Patient satisfaction with BR was assessed using the Alderman scale, a validated scale with breast-specific, well-described subcriteria to characterize clinical and esthetic outcomes of BR [34]. The scale consists of 7 items. Participants were asked to respond to each item using a 5-point Likert scale ranging from ‘strong dissatisfaction’ to ‘strong satisfaction’. The scale produces a total score ranging from 7 to 35 points, in which higher score indicates a higher level of satisfaction.

### Statistical analysis

The demographic characteristics and clinical variables for the patient population were summarized as means with standard deviations (SD) for continuous variables and as numbers with valid percentages for categorical variables. A mean input method was employed for missing data. The independent samples t-test was used to verify differences in populations for continuous variables and the chi-square test was used to calculate differences for categorical variables.

In the primary analysis, a multiple logistic regression model was employed to explore psychosocial predictors of the patients’ motivation for BR. Potential predictors (including baseline NBI, RSES, PHQ-9, and GAD-7 scores) were categorized and subjected to the model with the acceptance of BR as a dependent variable. The reported OR with 95% CI and p-values is the product of multivariate analyses adjusting for potential demographic and medical variables with p-values < 0.05 from the simple bivariate analyses, and confounders derived from previous findings (including age, time since mastectomy, education level and insurance).

In the secondary analysis, a multivariate linear regression model was employed to explore psychosocial outcomes (including baseline measures and changes in NBI, RSES, PHQ-9, and GAD-7 scores) associated with patient satisfaction scores on the Alderman scale. Confounders derived from previous findings (including surgical types, postoperative complications, pain and scarring) were entered into the multiple model for adjustment. Adjusted R-squares were calculated to indicate the goodness and fitness of the model.

All data were processed using the statistical package SPSS 17.0 (SPSS Inc., Chicago). A p-value of 0.05 was considered to be significant.

## Results

### Baseline characteristics

The clinical characteristics of the 264 participants are shown in Table 1. The mean age was 44.7 years old (SD = 7.2, range 24–62 years), and the mean time since mastectomy was 5.7 years (SD = 4.4, range 1–23 years). Most of the participants were high school educated or above (81.4%), stably employed (62%), married or living in a marriage-like situation (77%) and had children (83%) and an annual household income greater than RMB100000 (75%), while less than 5% had insurance covering BR. With respect to spousal attitude in the married group, 58% of husbands recommended their wife undergo BR, 14% recommended they do not, while the rest held a neutral attitude. Twelve percent of the respondents presented low self-esteem (RSES scores < 25), 36% fulfilled the criteria for mild to moderate degree of depression (PHQ-9 scores between 5 to 10), 51% presented mild to severe body image disturbance (NBI scores ≥ 6), while presence of anxiety symptoms (GAD-7 scores ≥ 5) was rare (5%) overall.

**Table 1:** Demographic and psychological characteristics of the sample and comparisons between the BR group and no-BR group.

Variables	Total	BR	No-BR	P-value
**Age at baseline visit** *n = 264 (%)*				
≤ 45	143 (54.2)	82 (65.1)	61 (44.2)	
> 45	121 (45.8)	44 (34.9)	77 (55.8)	**< .001**
Mean (sd)	44.7 (7.2)	43.3 (7.0)	46.0 (7.2)	**.002**
**Years since mastectomy** *n = 264 (%)*				
≤ 5	166 (62.9)	87 (69.0)	79 (57.2)	
> 5	98 (37.1)	39 (31.0)	59 (42.8)	**.047**
Mean (sd)	5.7 (4.4)	5.4 (4.6)	6.0 (4.1)	.284
**Laterality** *n = 264 (%)*				
Bilateral	11 (4.2)	4 (3.5)	7 (5.1)	
Unilateral	253 (95.8)	122 (96.5)	131 (94.9)	.441
**Education level** *n = 264 (%)*				
Primary school	55 (20.9)	20 (15.9)	35 (25.4)	
High school	64 (24.2)	27 (21.4)	37 (26.8)	
College or above	145 (54.9)	79 (62.7)	66 (47.8)	**< .001**
**Employment status** *n = 264 (%)*				
Unemployed/retired	100 (37.9)	43 (33.3)	57 (41.3)	
Employed	164 (62.1)	83 (66.7)	81 (58.7)	.230
**Marital status** *n = 238 (%)*				
Single/divorced	34 (14.3)	12 (10.3)	22 (18.2)	
Married/cohabited	204 (85.7)	105 (89.7)	99 (81.8)	.124
**Annual household income** *n = 256 (%)*				
< RMB 100000	78 (30.5)	34 (27.6)	44 (33.1)	
≥ RMB 100000	178 (69.5)	89 (72.4)	89 (66.9)	.345
**Children** *n = 264 (%)*				
None	46 (17.4)	19 (16.7)	27 (19.6)	
One or more	218 (82.6)	107 (83.3)	111 (80.4)	.708
**Insurance for BR** *n = 264 (%)*				
No	252 (95.5)	117 (92.1)	135 (97.8)	
Yes	12 (4.5)	9 (7.9)	3 (2.2)	.053
**Partner’s attitude** *n = 194 (%)*				
Negative	29 (14.2)	9 (8.7)	20 (22.0)	
Neutral	53 (28.1)	25 (24.3)	28 (30.8)	
Positive	112 (57.7)	69 (67)	43 (47.2)	**.001**
**Lymph node dissection** *n = 264 (%)*				
No	75 (28.4)	34 (27)	41 (29.7)	
Yes	189 (71.6)	92 (73)	97 (70.3)	.624
**Radiotherapy** *n = 264 (%)*				
No	154 (58.3)	80 (63.5)	70 (50.7)	
Yes	110 (41.7)	46 (36.5)	68 (49.3)	**.036**
**Chemotherapy** *n = 264 (%)*				
No	70 (26.5)	33 (26.2)	30 (21.7)	
Yes	194 (73.5)	93 (73.8)	108 (78.3)	.397
**Hormonal therapy** *n = 264 (%)*				
No	179 (67.8)	78 (61.9)	94 (68.1)	
Yes	85 (32.2)	48 (38.1)	44 (31.9)	.290
**NBI score** *n = 264 (%)*				
≤5	195 (73.9)	77 (61.1)	118 (85.5)	
6–9	69 (26.1)	49 (38.8)	20 (14.5)	**< .001**
Mean (sd)	5.7 (1.2)	6.0 (1.2)	5.3 (1.0)	**< .001**
**RSES score** *n = 264 (%)*				
≤ 24	31 (11.7)	23 (18.3)	8 (5.8)	
25–29	120 (45.5)	66 (52.4)	54 (39.1)	
≥ 30	113 (42.8)	37 (29.3)	76 (55.1)	**< .001**
Mean (sd)	28.6 (3.4)	27.5 (3.0)	29.6 (3.3)	**< .001**
**PHQ-9 score** *n = 264 (%)*				
0–4	170 (64.4)	83 (65.9)	87 (63)	
≥ 5	94 (35.6)	43 (34.1)	51 (37)	.632
Mean (sd)	3.7 (1.7)	3.7 (1.7)	3.7 (1.6)	.988
**GAD-7 score** *n = 264 (%)*				
0–4	251 (95.1)	118 (93.7)	133 (96.4)	
≥ 5	13 (4.9)	8 (6.3)	5 (3.6)	.307
Mean (sd)	1.7 (1.3)	1.8 (1.4)	1.6 (1.3)	.349

NBI, negative body image; RSES, Rosenberg Self-Esteem Scale; GAD-7, Generalized Anxiety Disorder seven-item; PHQ-9, Patient Health Questionnaire nine-item; sd, Standard Deviation; p value in bold when less than 0.05.

### Predictors of decision making for BR

Although all participants showed various level of initial interest in BR at the baseline interview, only 47% (126/264) finally underwent BR, while 53% (138/264) opted not to and had no plan for it in the near future. Table 1 compares the demographic and medical data and baseline psychological variables between the BR and no-BR groups. Factors significantly associated with the acceptance of BR were younger age (> 45 years old) (P < 0.01), shorter time span from mastectomy (< 5 years) (P < 0.05), higher education level (P < 0.01), no radiotherapy (P = 0.04), positive recommendation from spouse (P < 0.01), lower self-esteem (P < 0.01) and more severe body image disturbance (P < 0.01).

The potential psychosocial predictors of BR and confounders were combined in a multivariate logistic regression model. Results showed that low self-esteem, an RSES score ≤ 24 (vs. RSES score ≥ 30, OR = 2.943; 95% CI = 1.000–8.667; P < 0.05), high body disturbance, an NBI score ≥ 6 (vs. NBI score < 6, OR = 2.949; 95% CI = 1.443–6.025; P < 0.01), and having a college education (vs. primary school education or high school education, OR = 2.457 or 2.463; 95% CI = 1.263–4.762 or 1.079–5.649; P < 0.01 or P < 0.05) remained significant in the fully adjusted model. (Table 2)

**Table 2:** Multivariate logistic regression model identifying predictors of BR.

	Adjusted	
Variables	OR (95% CI)	P-value
**Age at baseline visit**		
≤ 45		
> 45	.585 (.295–1.166)	.126
**Years since mastectomy**		
≤ 5		
> 5	.948 (.502–1.791)	.870
**Education level**		**.015**
College or above		
High school	.406 (.177-.927)	**.016**
Primary school	.407 (.210-.792)	**.008**
**Insurance for BR**		
No		
Yes	3.610 (.839–15.535)	.085
**Radiotherapy**		
No		
Yes	.833 (.464–1.494)	.540
**NBI score**		
≤ 5		
6–9	2.949 (1.443–6.025)	**.003**
**RSES score**		**.027**
≥ 30		
25–29	2.055 (1.145–3.687)	.**016**
≤ 24	2.943 (1.000–8.667)	**.048**
**PHQ-9 score**		
0–4		
≥ 5	.549 (.295–1.021)	.058
**GAD-7 score**		
0–4		
≥ 5	2.072 (.540–7.952)	.289

NBI, negative body image; RSES, Rosenberg Self-Esteem Scale; GAD-7, Generalized Anxiety Disorder seven-item; PHQ-9, Patient Health Questionnaire nine-item; p value in bold when less than 0.05.

Additional exploratory analysis was conducted to examine associations within the married group. We re-ran the multivariate model with spousal attitude entered in place of marital status. Results showed that an RSES score ≤ 24 (vs. RSES score ≥ 30, OR = 5.130; 95% CI = 1.292–20.376; P < 0.05), an NBI score ≥ 6 (vs. NBI score < 6, OR = 2.525; 95% CI = 1.060–6.014; P < 0.05), and a positive spousal attitude toward BR (vs. a negative spousal attitude, OR = 3.245; 95% CI = 1.124–9.369; P < 0.05) remained significant predictors of BR. (Table 3)

**Table 3:** Multivariate logistic regression model identifying predictors of BR (married group).

	Adjusted	
Variables	OR (95% CI)	P-value
**Age at baseline visit**		
≤ 45		
> 45	.634 (.276–1.456)	.283
**Years since mastectomy**		
≤ 5		
> 5	.804 (.381–1.700)	.571
**Education level**		.098
College or above		
High school	.604 (.222–1.644)	.323
Primary school	.428 (.197-.928)	**.032**
**Spousal attitude**		**.047**
Negative		
Neutral	1.759 (.589–5.254)	.312
Positive	3.245 (1.124–9.369)	**0.030**
**Insurance for BR**		
No		
Yes	3.156 (.564–17.665)	.191
**Radiotherapy**		
No		
Yes	.890 (.444–1.783)	.742
**NBI score**		
≤5		
6–9	2. 525 (1.060–6.014)	**.036**
**RSES score**		**.032**
≥ 30		
25–29	2.028 (1.005–4.094)	**.048**
≤ 24	5.130 (1.292–20.376)	**.020**
**PHQ-9 score**		
0–4		
≥ 5	.353 (.219–1.104)	.053
**GAD-7 score**		
0–4		
≥ 5	3.335 (.653–17.030)	.148

NBI, negative body image; RSES, Rosenberg Self-Esteem Scale; GAD-7, Generalized Anxiety Disorder seven-item; PHQ-9, Patient Health Questionnaire nine-item; p value in bold when less than 0.05.

In order to test for sensitivity to variable categorization, the model selection was replicated using continuous variables, which led to substantially unmodified results (data not shown).

### Postoperative psychosocial outcome measures

Of the BR patients, 114 patients responded, for a response rate of 90 percent. When comparing nonresponders (n = 12) to the responders (n = 114), there were no significant differences in the following variables: age, education, employment status, marital status, and postoperative complication. The mean follow-up time for the respondents was 6.9 (SD = 1.7) months. The mean satisfaction score on the Alderman scale was 27.6 (SD = 2.3). The mean postoperative RSES score was 29.3 (SD = 2.6), which was significantly increased from the mean score of 27.3 (SD = 3.1) at baseline (P < 0.01). The mean NBI score decreased from 3.7 (SD = 1.8) at baseline to 2.8 (SD = 1.3) postoperatively (P < 0.01), and the mean PHQ-9 score decreased from 6.1 (SD = 1.2) at baseline to 4.5 (SD = 0.9) postoperatively (P < 0.01), whereas no significant difference was seen in the mean GAD-7 score. (Table 4)

**Table 4:** Psychosocial outcomes of BR group before and 6 months after surgery.

	Pre-	Post—	
Variables	Mean (SD)	Mean (SD)	P-value
**NBI score**	6.1 (1.2)	4.5 (.9)	**< .001**
**RSES score**	27.2 (3.1)	29.3 (2.6)	**< .001**
**PHQ-9 score**	3.7 (1.8)	2.8 (1.3)	**< .001**
**GAD-7 score**	1.8 (1.4)	1.7 (1.2)	.107
**Alderman score**	—-	27.6 (2.3)	

SD, Standard Deviation; NBI, negative body image; RSES, Rosenberg Self-Esteem Scale; GAD-7, Generalized Anxiety Disorder seven-item; PHQ-9, Patient Health Questionnaire nine-item; p value in bold when less than 0.05.

### Variables associated with satisfaction with BR

Table 5 summarizes the clinical and psychological outcomes associated with patient satisfaction scores on the Alderman scale, and provides the corresponding coefficients estimated by multiple linear regression models. Several features remained significant in the fully adjusted model including; the preoperative NBI score (coefficient = -0.634; SE = 0.202; P < 0.01), the postoperative change from baseline NBI score (coefficient = -0.619; SE = 0.208; P < 0.01), the change from baseline RSES score (coefficient = 0.281; SE = 0.083; P < 0.01), the change from baseline PHQ-9 score (coefficient = -0.523; SE = 0.209; P < 0.05), the pain score (coefficient = -0.555; SE = 0.222; P < 0.05), and the scarring score (coefficient = -0. 594; SE = 0.161; P < 0.01). The model explained 47.1% of the variance in patient satisfaction with an F-ratio of 9.395.

**Table 5:** Multiple linear regression analysis for factors associated with satisfaction with BR.

	Adjusted		
Variables	Coefficient	SE	P-value
***Continuous***			
**Scarring score**	-.594	.161	**< .001**
**Pain score**	-.555	.222	**.014**
**NBI score**			
Baseline	-.634	.202	**.002**
Change	-.619	.208	**.004**
**RSES score**			
Baseline	.018	.074	.804
Change	.281	.083	**.001**
**PHQ-9 score**			
Baseline	-.192	.157	.224
Change	-.523	.209	**.014**
**GAD-7 score**			
Baseline	.130	.175	.461
Change	.111	.190	.559
***Categorical***			
**Complications after BR**			
No	-	-	
Yes	-.131	.429	.760
**Type of BR**			
DIEP flap	**-**	**-**	
LD flap plus implant	-.589	.617	.342

SE, standard error; CI, confidence interval; NBI, negative body image; RSES, Rosenberg Self-Esteem Scale; GAD-7, Generalized Anxiety Disorder seven-item; PHQ-9, Patient Health Questionnaire nine-item.

## Discussion

Asian BC survivors are less likely to undergo BR after mastectomy compared with their Caucasian counterparts, even when there is the same access to medical financial services available [10,17,35,36]. The rate of BR in the Chinese population is estimated to be even lower [37].

In accordance with the literature that emphasized concern about body image as a primary reason for women pursuing reconstructive surgeries [38–40], we identified negative body image as an independent and stable predictor of BR, even after control for socio-demographic variables. Over eighty percent of our participants experienced two or more body image problems at least some of the time, or at least one problem much of the time. For women, body image means feeling feminine and attractive [41]. Culturally, the influence of the Oriental virtue of modesty, was suggested to mean that traditional Chinese women placed less emphasis on breasts in maintaining their feminine identity compared to facial appearance and virtuous behavior. Many traditional Chinese women had been brought up to believe that it was inelegant and inappropriate to publicly expose the curve of their breasts [42]. However, in the current study, ‘feeling less feminine and sexually desirable’ were common serious problems reported by our participants, which was similar to the Western study by Fobair et al. [31]. It seems that modern Chinese women with higher education level and higher economic freedom express stronger concerns about breast loss impacting on their feminine identity and marital relationships with their partners.

As hypothesized, lower self-esteem after mastectomy was related to an increased motivation for women to undergo BR. The finding was consistent with the previous Western study by Goldberg et al. [43]. With the changes in social roles and the elevation of social status, more women in mainland China are pursuing plastic surgeries for boosting their body confidence than ever before [44]. Female BC patients with mastectomy may have low self-esteem because of altered appearance, changes in social identity, and uncertainty of future [45]. Low self-esteem may aggravate the impairment of their social function by holding them back from their regular social plans and social circle [46]. Seeking reconstructive surgeries can be viewed as an effort to rebuild their self-esteem and restore their social ties.

Contrary to what was expected, there were few BR patients that suffered from severe depression or anxiety before reconstruction. Some prior studies demonstrated that depression and anxiety symptoms after mastectomy were the motivation for some patients to opt for BR [47,48]. Other researchers had concluded that depressive symptoms might lead to anhedonia, impaired appreciative ability, and impairment in motivation on reward-based decision-making [49]. In this study, no definite predictive strength of these two variables for BR was confirmed. Similar results were observed in a recent cross-sectional cohort study of 216 Polish women by Zycinska et al. [50] They concluded that depression and anxiety, as negative aspects of well-being following mastectomy, had less direct effects on the patients’ intention to undergo BR.

Consistent with what was found in the Western studies [14,18], education level was identified as a significant demographic covariate in the multiple regression model, indicating higher likelihood of BR in women with a college diploma compared to women with lower levels of education. There was also a trend toward a higher rate of BR in women younger than 45 years old or within the first five-year after mastectomy, yet these factors were not significant in the multivariate model. Interestingly, in the subanalysis of 204 married women, the husband’s positive attitude toward BR was identified as an independent predictor of surgical acceptance. In this position, Chinese female patients might be more susceptible to the influence of their husband in a subconscious attempt to maintain family harmony.

In assessing satisfaction with BR, a mean score of 27.6 on the Alderman scale was observed, which was higher than observed in previous Western studies [51,52] indicating these patients were more satisfied with the outcome of BR. It is noteworthy that our study samples consisted of a higher proportion of women undergoing microsurgical reconstruction. BR with autologous tissue flaps has been proved to achieve a more ideal aesthetic outcome than BR with implants, as it provides a more natural appearance closely resembling the contour and feel of the unaffected breast [53]. In addition, most of our patients underwent delayed BR at least 6 months after mastectomy. It is predictable that a woman having consciously experienced the mutilating effect of mastectomy would have higher appreciation for the outcome of delayed BR than a woman with immediate BR who compares the reconstructed breast with her natural breast.

Significant improvement in self-esteem, body image, and a significant reduction of depression were observed in patients undergoing BR at 6 months postsurgery. Multivariate regression analyses revealed that most baseline measures of severity were not related to satisfaction ratings except for the preoperative NBI score, which was negatively correlated with the postoperative Alderman score. By contrast, postoperative scores on the NBI, RSES, and PHQ-9 scales were generally at least moderately correlated with satisfaction ratings after adjustment for the potential clinical confounders. Similar results were obtained in the observational study by Dawson et al. [54] who demonstrated that postoperative scores rather than preoperative scores for social-psychological scales were correlated with patient satisfaction with their surgical outcomes. Our findings provide evidence for a strong connection between BR patient satisfaction and postoperative alleviation of negative psychological impact of breast loss.

### Limitation

The present study has limitations that should be mentioned. First, a selection bias might be present because we relied on a convenient sample of outpatient populations already interested in BR, which might not represent the general population of Chinese BC survivors. Second, since our institution is a regional referral center for microsurgical reconstruction, most patients transferred to our department were not suitable for simple implant BR because of poor skin condition. Thus, we were unable to compare the subgroups of patients with autologous BR to patients with a simple implant BR for the studied endpoints. Third, a second assessment of psychosocial outcomes for the no-BR patients was limited because of loss to follow-up. Some studies have proposed that psychosocial functioning after mastectomy might be generally improved over time, regardless of whether a reconstruction was performed [55]. This is an interesting concept which might partly explain the higher level of self-esteem and body image for the no-BR group patients in this study, since they experienced longer average times since mastectomy than the patients in the BR group. Based on this hypothesis and our conclusion, they might have opted for BR had it been available earlier, but with time their body image and self-esteem were improved and their motivation for BR was reduced. However, this hypothesis cannot be evaluated in the present study and would need further investigation.

## Conclusion

This study demonstrates that lower self-esteem and negative body image are independent psychosocial predictors of delayed BR for Chinese BC survivors. In addition, psychological benefits following reconstruction, including improvements in self-esteem, body image, and reduction in depression are significantly associated with patient postoperative satisfaction.
